# Genome-wide association study and genomic prediction of white rust resistance in USDA GRIN spinach germplasm

**DOI:** 10.1093/hr/uhac069

**Published:** 2022-03-23

**Authors:** Ainong Shi, Gehendra Bhattarai, Haizheng Xiong, Carlos A Avila, Chunda Feng, Bo Liu, Vijay Joshi, Larry Stein, Beiquan Mou, Lindsey J du Toit, James C Correll

**Affiliations:** 1 Department of Horticulture, University of Arkansas, Fayetteville, AR 72701, USA; 2 Department of Horticultural Sciences, Texas A&M AgriLife Research and Extension Center, Weslaco, TX 78596, USA; 3 Department of Plant Pathology, University of Arkansas, Fayetteville, AR 72701, USA; 4 Texas A&M AgriLife Research and Extension Center, Uvalde, TX 77801, USA; 5 Crop Improvement and Protection Research Unit, USDA-ARS, Salinas, CA 93905, USA; 6 Washington State University, Mount Vernon, WA 98273, USA

## Abstract

White rust, caused by *Albugo occidentalis*, is one of the major yield-limiting diseases of spinach (*Spinacia oleracea*) in some major commercial production areas, particularly in southern Texas in the United States. The use of host resistance is the most economical and environment-friendly approach to managing white rust in spinach production. The objectives of this study were to conduct a genome-wide associating study (GWAS), to identify single nucleotide polymorphism (SNP) markers associated with white rust resistance in spinach, and to perform genomic prediction (GP) to estimate the prediction accuracy (PA). A GWAS panel of 346 USDA (US Dept. of Agriculture) germplasm accessions was phenotyped for white rust resistance under field conditions and GWAS was performed using 13 235 whole-genome resequencing (WGR) generated SNPs. Nine SNPs, chr2_53 049 132, chr3_58 479 501, chr3_95 114 909, chr4_9 176 069, chr4_17 807 168, chr4_83 938 338, chr4_87 601 768, chr6_1 877 096, and chr6_31 287 118, located on chromosomes 2, 3, 4, and 6 were associated with white rust resistance in this GWAS panel. Four scenarios were tested for PA using Pearson’s correlation coefficient (r) between the genomic estimation breeding value (GEBV) and the observed values: (1) different ratios between the training set and testing set (fold), (2) different GP models, (3) different SNP numbers in three different SNP sets, and (4) the use of GWAS-derived significant SNP markers. The results indicated that a 2- to 10-fold difference in the various GP models had similar, although not identical, averaged r values in each SNP set; using GWAS-derived significant SNP markers would increase PA with a high r-value up to 0.84. The SNP markers and the high PA can provide valuable information for breeders to improve spinach by marker-assisted selection (MAS) and genomic selection (GS).

## Introduction

Spinach (*Spinacia oleracea* L.) is one of the important vegetable crops in the world economically, which was estimated to average $490 million (fresh and for processing) per year during 2018–20 in the United States (US), with 97% of the value for the fresh market [1]. Spinach is considered a “super food” due to a high concentration of phytonutrients and other health-promoting compounds, including vitamin A and C, carotenoid, lutein, folate, calcium, iron, and antioxidants [2, 3]. In the US, spinach has become very popular during the past two decades as healthy-conscious consumers have increased the consumption of leafy vegetables. To meet the greater demand for spinach, commercial production has evolved into high density (up to 10 million/ha) planting, year-round production cycles, overhead sprinkler irrigation systems, high fertilizer application, and expanded production areas; all of them create an environment conducive for the development of diseases. Several diseases reduce yield and quality individually or in combination, thus posing serious challenges to commercial spinach production. Spinach suffers from many diseases while downy mildew, white rust, Fusarium wilt, Stemphylium leaf spot, and anthracnose leaf spot are the most devastating and economically important diseases of spinach.

White rust of spinach is caused by *Albugo occidentalis*, an obligate oomycete that can reduce yield and quality [3–8]. White rust has been an endemic throughout the central and eastern US for many years but has also been reported in other parts of the world, including Greece [9], Mexico [10], and Turkey [11]. The persistent appearance of this disease and its expansion into wider geographic areas pose a significant challenge to the spinach industry in the US and world. If this pathogen is introduced into major US production areas in California and Arizona that produce over 90% of the fresh market product, it would be devastating to the US spinach industry as the vast majority of the cultivars adapted to these areas have little to no resistance incorporated. Resistance to white rust has been previously found in USDA spinach germplasm and breeding lines [4, 7, 12–14]. High levels of resistance to white rust have been reported in the spinach varieties developed at the spinach breeding program of University of Arkansas as this disease was a primary breeding goal [3]; thus, the Arkansas germplasm has been used as a source of resistance to transfer white rust resistance into several commercial cultivars. However, even this resistance material can suffer severe infection when conditions are highly conducive for the disease development [7, 12–14]. Yet, there is little information regarding the genetics of white rust resistance in spinach. Thus it is necessary to routinely evaluate and identify new spinach resources for white rust resistance for the development of cultivars with improved resistance. So far, only quantitative resistance has been found and utilized as no major genes have been reported for white rust resistance [5].

The publication of reference genomes and other new assemblies [15–19] has made genome-wide variant discovery in the germplasm panel and genome-wide association studies (GWAS) possible in spinach. With the decreasing genotyping cost in recent years and advanced statistical methods, GWAS and genomic selection (GS) approaches are commonly utilized to improve complex genetic traits in crops. GWAS, based on the genotyping and phenotyping of a natural germplasm population and high-density markers, has been employed to map simple to complex traits and identify candidate genes in many crops [20–22]. GWAS has been used in spinach for many traits, including surface texture, edge shape, and petiole color [23]; bolting, tallness, and erectness [24]; leafminer resistance [25]; oxalate concentration [26]; Verticillium wilt resistance [27], *Stemphylium* leaf spot resistance [28]; mineral nutrient contents [29]; white rust resistance [30]; growth habit [31]; anthracnose resistance [32]; and downy mildew resistance [33–36]. The identification of single nucleotide polymorphism (SNP) markers for the traits has provided valuable molecular tools for breeders to develop spinach cultivars more efficiently. It has been demonstrated that quantitative trait of white rust resistance in spinach can be screened under field conditions and is correlated with quantitative resistance to downy mildew [4, 37]. Using a panel of 267 spinach accessions with 6111 SNPs, Awika et al. (2019) [30] conducted GWAS analysis for white rust resistance and identified 448 minor alleles (SNPs) associated with white rust disease severity, which maybe be utilized in the selection for resistant plants.

Genomic prediction (GP) is emerging as a promising tool to improve the efficiency and speed of plant breeding. So far, GP has been reported in several crops [38–48] for various traits. Genomic estimation breeding value (GEBV) is a key step in GS. Several approaches have been proposed to determine GEBV, such as Bayesian methods (Bayes A, Bayes B, Bayes LASSO, and Bayes ridge regression) and BLUP methods (RR-BLUP, gBLUP, and cBLUP). The GS approaches have been adopted for variety of traits in various crops [49–55]. All articles reported the prediction accuracy (PA) estimates using the Pearson’s correlation coefficient (r) between the observed phenotypic values and the predicted GEBV for each trait in a validation set using several different models.

Currently, USDA-GRIN (Germplasm Resources Information Network) has approximately 400 spinach accessions, which were originally collected from 33 countries and represent a diverse germplasm collection. The overall objectives of this study were to evaluate USDA spinach germplasm for white rust resistance under the field conditions and to identify resistance-associated SNP markers through GWAS to conduct marker-assisted selection and genomic selection for white rust resistance.

## Materials and methods

### Plant materials (genome-wide association panel)

Three hundred forty-six spinach accessions of the USDA-GRIN spinach germplasm collection were phenotyped for white rust disease and genotyped by using whole-genome resequencing in this study (Table S1 where S signifies supplementary). The accessions in this GWAS panel were originally collected from 32 countries, with a majority (81.5%) from ten countries: Turkey (n = 107), United States (US) (n = 55), China (n = 25), North Macedonia (n = 22), Afghanistan (n = 20), Iran (n = 15), Belgium (n = 10), India (n = 10), Syria (n = 10), and Hungary (n = 8) (Table S1).

### White rust phenotyping

The 346 spinach accessions were evaluated for white rust resistance in the Del Monte White Rust Nursery, Crystal City, Texas, during the three winter seasons of 2015–16, 2016–17, and 2017–18. The nursery is known to have high white rust disease pressure over the three decades. The field experiments were performed in a randomized complete block (RCB) design with two replications. In each block, each accession was planted in a 10-feet long row, three feet between rows, and 4-inch between plants within the row. Thus, there were about 30 plants in each row with 60 plants of each accession for evaluation each year. White rust disease was evaluated under natural disease pressure without introducing external inoculum. A susceptible cultivar, Viroflay, was planted as a spreader row on both sides of the tested genotypes.

White rust disease was rated using a scale of 0 to 10 whereby a 0 = no disease, 1 = <1% of the total leaf area covered with white rust infection, 2 = 1–10%, 3 = 11–20%, 4 = 21–30%, 5 = 31–40%, 6 = 41–50%, 7 = 51–60%, 8 = 61–70%, 9 = 71–80%, and 10 = > 80% infected leaf area. After 65–70 days from planting, around ten plants of each genotype were scored for disease severity by estimating the proportion of total leaf area canopy with symptoms (chlorotic and necrotic lesions) and signs (sporulation and pustules). The white rust disease severity was recorded 2–3 times each season.

The 2017–2018 winter season trial had high white rust disease pressure among the three years of evaluations, while the disease severity was lower in the other two years (data not shown), thus only disease severity data from the 2017–18 winter season were reported in this study. The white rust response data of the 346 spinach accessions were analyzed for the analysis of variance (ANOVA) with the general linear models (GLM) in JMP Genomics 9 (SAS Institute, Cary, NC). Multiple comparisons among individual accessions were performed using the student T-test at *α* = 0.05, and mean, range, standard deviation (SD), standard error (SE), and coefficient of variation (CV) of disease severity were computed. Distribution of white rust disease across accessions was drawn and the mean disease rating of each accession was used as the phenotypic data for GWAS.

### Genotyping

DNA was extracted from fresh leaves bulked from 5–10 plants for each genotype. Qualified DNA for each sample was sheared randomly into 350-bp fragments by Covaris Ultrasonic Processor before sequencing. The construction of the DNA libraries followed the process of end repairing, adding A tails, purification, PCR amplification and library qualification [56]. The DNA libraries were pair-end sequenced by whole-genome resequencing (WGR) technology at 10x spinach genome size coverage generating about 10 Gb sequence data for each sample using Illumina NovaSeq Sequencer machine at BGI (https://www.bgi.com/). The spinach genome of Sp75 [18, 57] available at SpinachBase was used as a reference to map the WGR data of the 346 spinach genotypes using Burrows-Wheeler aligner software (BWA v0.7.8-r455 [58]). SAMtools (v 0.1.19-44 428 cd) [58] were utilized to sort the bam files and remove duplication reads. The program Picard (v 1.111) [58] was used to merge the bam files from the same sample, and the GATK software (v 3.5) [59] was chosen to detect and filter SNPs and InDels.

Around 16 million raw SNPs were identified in the 346 spinach genotypes. Filtering and keeping the SNPs with minor allele frequency (MAF) >2%, missing allele <30%, and heterogeneous rate < 50%, retained 2 357 260 SNPs distributed on six chromosomes (chr) that twere used in this study. There are 217 531 SNPs on chr 1; 239 902 SNPs on chr 2; 651, 097 SNPs on chr 3; 629, 147 SNPs on chr 4; 334 526 SNPs on chr 5; and 285 057 SNPs on chr 6.

### Principal component analysis (PCA) and genetic diversity

In this study, 8399 SNPs were randomly selected from the 2 357 260 SNPs: 1073 SNPs on chr 1; 1106 SNPs on chr 2; 2123 SNPs on chr 3; 1997 SNPs on chr 4; 1020 SNPs on chr 5; and 1080 SNPs on chr 6 (FigShare: https://doi.org/10.6084/m9.figshare.17283194). The selected set of SNPs was included in the principal component analysis (PCA) and genetic diversity analysis. PCA and genetic diversity were analyzed with GAPIT 3 (Genomic Association and Prediction Integrated Tool version 3) [54, 60] (https://zzlab.net/GAPIT/index.html;https://github.com/jiabowang/GAPIT3) by setting PCA = 2 to 10 and NJ tree = 2 to 10. Phylogenetic trees were drawn by using neighbor-joining (NJ) method.

### Association analysis

GWAS was performed in a two step process. In the first step, 2 357 260 SNPs were used to perform GWAS implementing single marker regression (SMR), GLM (PCA), and MLM (PCA + K) methods in TASSEL 5 [61]. However, we only used the 8399 randomly selected SNPs to estimate PCA and Kinship matrixes. PCA matrix was estimated with the PCA tool in TASSEL 5, setting covariance (alterative = correction) and the number of components = 2. Kinship (K) was estimated in TASSEL 5 by using Scald_IBS method. Based on GWAS analysis in TASSEL 5, there were 4836 SNPs with the logarithm of odds (LOD) [−Log_10_(P-value)] > 4.0 either in SMR, GLM, or MLM (We use LOD instead of –Log_10_(P-value) in this article.).

In the second step, 13 235 SNPs (4836 associated SNPs in the first step plus the randomly selected 8399 SNPs used for PCA and Kinship analysis) (FigShare: https://doi.org/10.6084/m9.figshare.17283194) distributed on the six spinach chromosomes (Fig. S1) were used to perform GWAS using the SMR, GLM, and MLM models in TASSEL 5 and several models in GAPIT 3 [54, 60] program. In GAPIT3, GWAS was performed using the general linear model (GLM), mixed linear model (MLM), compressed MLM (cMLM) [62], Settlement of MLM Under Progressively Exclusive Relationship (SUPER) [63], multiple-locus MLM (MLMM), fixed and random model circulating probability unification (FarmCPU) [64] and bayesian-information and linkage-disequilibrium iteratively nested keyway (BLINK) [65] models. In addition, a *t*-test was conducted for all 13 235 SNPs by using visual basic codes in Microsoft Excel 2016.

Multiple TASSEL and GAPIT models were used to find reliable and stable white rust resistance-associated SNP markers and candidate genes and QTL regions in spinach. The significant threshold of associations was calculated using Bonferroni correction of P-value with an α = 0.05 (0.05 / SNP number), and LOD value of 5.42 was used as significance threshold based on the 13 235 SNPs in this study.

### Candidate gene identification/detection

Genes were searched within 50 Kb on either side of significant SNPs of the spinach Sp75 genome annotation at the SpinachBase site (http://www.spinachbase.org/). Our emphasis was to find analogs of disease resistance genes near the significantly associated SNP markers.

### Genomic prediction for genomic selection of white rust resistance

The ridge regression best linear unbiased prediction (rrBLUP) method was used to perfrom GP using the rrBLUP package [66] in R Version 4.0.5. In addition, GP was conducted with gBLUP and cBLUP implemented in GAPIT package [54]; Bayesian models including Bayes A, Bayes B, Bayes LASSO (BL), and Bayes ridge regression (BRR) implemented in BGLR package [67]; and random forest (RF) model implemented in Random Forest R package [68] and support vector machines (SVM) [68] implemented in kernlab packages. GP using these packages has been reported in previous studies [49–53].

GP for white rust resistance was performed in 346 spinach accessions based on ratios of training / testing sets, number of SNPs, and GP models. (1) GP was performed using nine different ratios of training / testing sets, 2 fold (1:1), 3-, 4-, 5-, 6-, 7, 8-, 9-, and 10-fold (9:1) across three sets of SNPs: (i) all 13 235 SNPs, (ii) 40 SNP markers, and (iii) 9 SNP markers (GWAS-derived SNP markers). (2) Eight different SNP number sets from 9 SNPs to 4846 SNPs were used to estimate GP by BL for three SNP sets: (i) Set.13235SNP, (ii) Set.4836SNP_select, and (iii) Set.8839SNP.random. (3) GP was estimated with nine GP models, BA, BB, BL, BRR, SVM, RF, rrBLUP, gBLUP, and cBLUP, in cross-prediction for white rust resistance among seven SNP sets (all, 40 m, 9 m, 40r, 9r, 40rr, and 9rr). These seven SNP sets were selected based on results obtained and information provided in the result section. In addition, GWAS-derived SNP markers for GP were analyzed and discussed.

The PA for the tested models in this study was estimated by calculating the average Pearson’s correlation coefficient (r) between the GEBVs estimates from the training set and white rust phenotypic values in the validation set or testing set [40, 53, 69]. The training and testing sets were randomly generated 100 times; the average r-value was estimated; and distribution charts (boxplots) were drawn using the ggplot2 R package.

## Results

### Evaluation of white rust resistance

**Figure 1:**
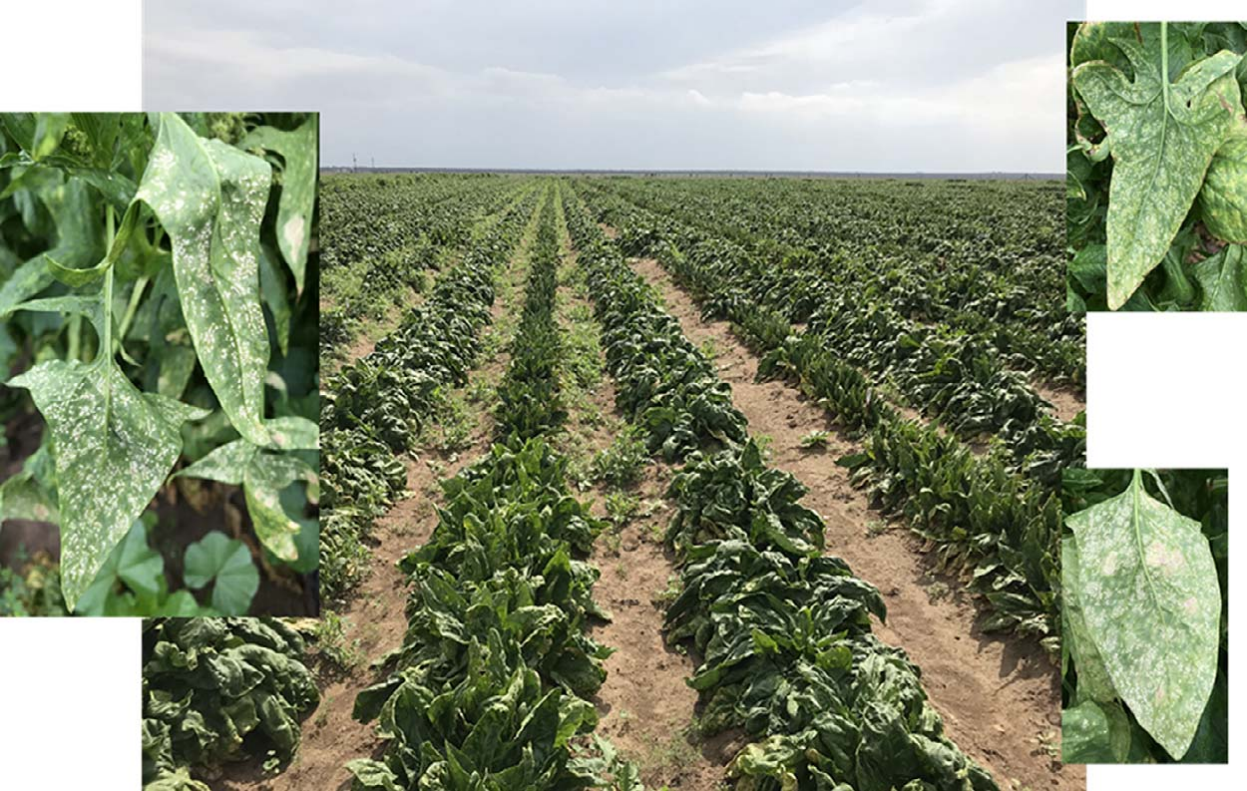
White rust field evaluation and leaf symptom

**Figure 2:**
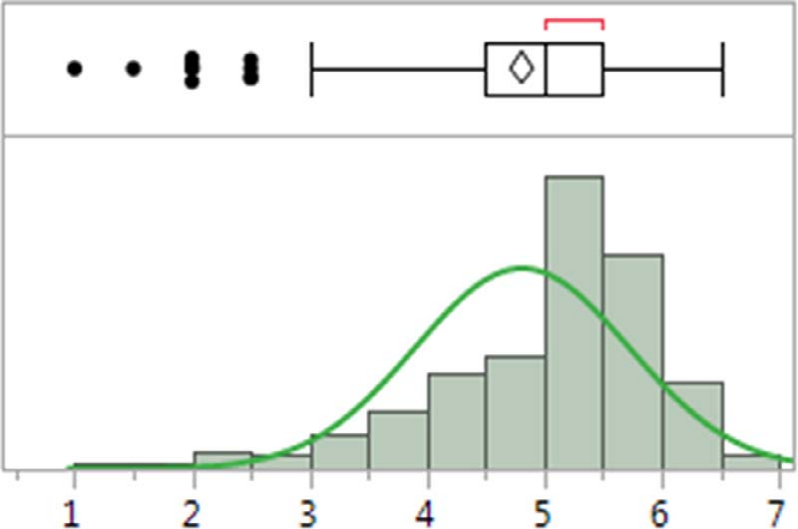
The distribution of white rust disease scale (0–10 scale) in 346 spinach lines

The white rust disease showed signs and symptoms on leaves, and the disease severity was recorded using the 0 to 10 disease scale (Fig. 1). The scale (0–10) in the 346 spinach accessions did not show a normal distribution but skewed toward a higher disease severity due to most material being highly susceptible (Fig. 2) in the association panel. The mean disease severity ranged from 1.0 to 6.5, averaged 4.8 with a standard deviation of 0.911 and the CV was 17.2%. The data showed an extensive range and variation of the white rust disease scale in the 346 accessions, confirming the suitability of the association panel for GWAS. The lines NSL 6098, PI 175311, PI 220686, PI 224959, PI 226671, PI 227045, and PI 648958 showed the highest white rust resistant levels with a score of 2.0 or less (Table 1 & S1), indicating their suitability as parents in breeding programs to develop white rust-resistant hybrids and cultivars.

**Table 1:** Top 23 spinach white rust resistant lines

**Line ID**	**ACCESSION**	**NAME**	**ORIGIN**	**Country**	**2-cluster**	**3-cluster**	**Group**	**WR Scale**
PI222270_Iran_Q1.G1_3	PI 222270	Esfenaj	Iran	Iran	Q1	G1	I	3.0
PI222838_Iran_Q1.G1_3	PI 222838	Esfenaj	Iran	Iran	Q1	G1	I	3.0
PI224959_Iran_Q1.G1_2	PI 224959	Cornell ID #4	Iran	Iran	Q1	G1	I	2.0
PI226671_Iran_Q1.G1_1	PI 226671	Cornell ID #10	Iran	Iran	Q1	G1	I	1.0
PI171858_Turkey_Q1.G1_3	PI 171858	Harlan 6652	Kastamonu, Turkey	Turkey	Q1	G1	I	3.0
PI173131_Turkey_Q1.G1_3	PI 173131	Cornell ID #87	Malatya, Turkey	Turkey	Q1	G1	I	3.0
PI171859_Turkey_Q1.G1_3	PI 171859	Harlan 6725	Samsun, Turkey	Turkey	Q1	G1	I	3.0
PI648951_UnitedStates_Q1.G1_3	PI 648951	Cornell ID #275	Maryland, United States	United States	Q1	G1	I	3.0
PI648957_UnitedStates_Q1.G1_3	PI 648957	76 X 71	Maryland, United States	United States	Q1	G1	I	3.0
PI648958_UnitedStates_Q1.G1_1.5	PI 648958	Cornell ID #286	Maryland, United States	United States	Q1	G1	I	1.5
PI648960_UnitedStates_Q1.G1_3	PI 648960	Cornell ID #288	Maryland, United States	United States	Q1	G1	I	3.0
PI648961_UnitedStates_Q1.G1_3	PI 648961	224 X 223	Maryland, United States	United States	Q1	G1	I	3.0
NSL6098_UnitedStates_Q1.G1_2	NSL 6098	Norfolk Savoy/ Bloomsdale	Virginia, United States	United States	Q1	G1	I	2.0
PI212119_Afghanistan_Q1.G1_3	PI 212119	Cornell ID #5	Afghanistan	Afghanistan	Q1	G1	I	3.0
PI207518_Afghanistan_Q1.G1_2.5	PI 207518	Cornell ID #30	Afghanistan	Afghanistan	Q1	G1	I-outlier	2.5
PI220686_Afghanistan_Q1.G1_2	PI 220686	Palek	Afghanistan	Afghanistan	Q1	G1	I-outlier	2.0
PI211632_Afghanistan_Q1.G1_2.5	PI 211632	Cornell ID #35	Afghanistan	Afghanistan	Q1	G1	I-outlier	2.5
PI212120_Afghanistan_Q1.G1_2.5	PI 212120	Cornell ID #6	Afghanistan	Afghanistan	Q1	G1	I-outlier	2.5
PI648949_China_Q2.G3_3	PI 648949	II9A0323	Beijing, China	China	Q2	G3	II	3.0
PI433210_China_Q2.G3_2.5	PI 433210	498	China	China	Q2	G3	II	2.5
PI165994_India_Q2.G2_3	PI 165994	Palak	India	India	Q2	G2	II	3.0
PI175311_India_Q2.G2_2	PI 175311	Palak	India	India	Q2	G2	II	2.0
PI227045_Iran_Q2.G2_2	PI 227045	Cornell ID #201	Iran	Iran	Q2	G2	II	2.0

### Genetic diversity among white rust-resistant lines

Among the 346 spinach accessions, 23 showed white rust resistance with a rate 3 or below (Table 1), indicating that the 23 spinach accessions can be used as parents to develop new spinach cultivars or lines for white rust resistance in breeding. Five of the 23 accessions were originally collected from Afghanistan; two from China; two from India; five from Iran; three from Turkey; and six from United States (Table 1), indicating that the white rust resistance was mainly distributed among Asia and U.S. accessions in this study.

**Figure 3:**
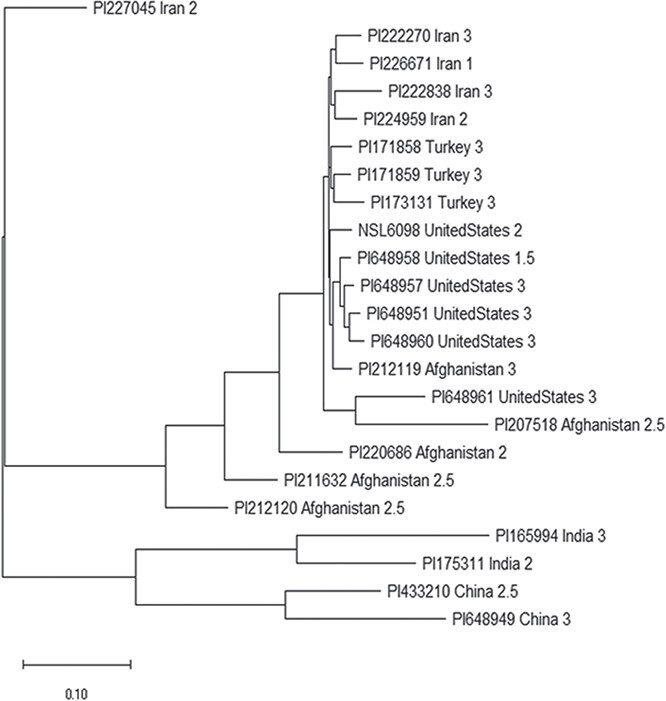
The phylogenetic tree among 23 spinach accessions of white rust resistance drawn by Mega 7. In the tree, the taxon name consists of the spinach accession ID, the accession original country, and the white rust scale. For the taxon name, PI648949 China 3 - PI648949, originally collected from China, and the white rust scale is 3.

The genetic diversity analysis among the 23 accessions showed that (1) the accessions from the same country were located at neighbor each other with less genetic distance in the phylogenetic tree in most cases; (2) two clusters were grouped: the five accessions, PI 227045, PI 165994, PI 175311, PI 433210 and PI 648949 from Iran, China and India as one group, and other 18 accessions as another group; and (3) In group I, the four accessions, PI 207518, PI 220686, PI 211632, and PI 212120 had different genetic base from others as I-outlier (Fig. 3, Table 1). The phylogenetic analysis will provide information on how to utilize these white rust-resistant accessions.

### PCA and phylogenetic analysis

**Figure 4:**
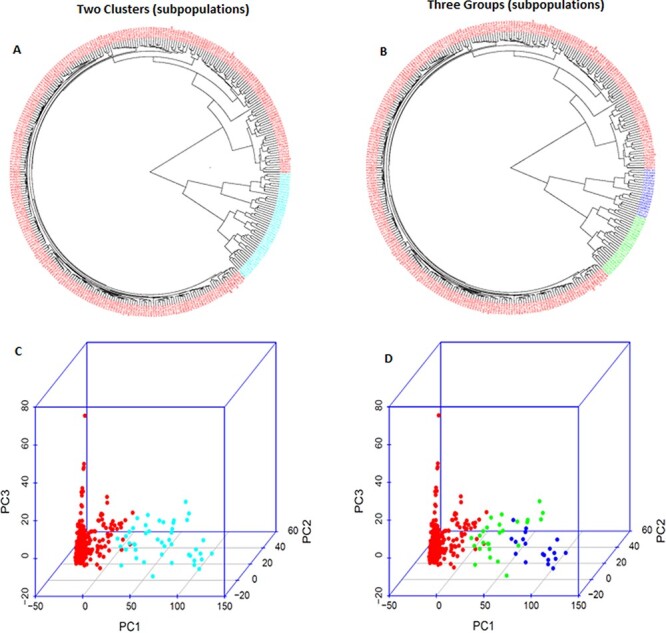
Population genetic diversity analysis in the association panel consisted of 346 USDA spinach germplasm accessions. Phylogenetic trees drawn by neighbor-joining (NJ) method in (A) two sub-population and (B) three sub-population, and 3D graphical plot of the principal component analysis (PCA) in (C) two sub-population and (D) three sub-population drawn by GAPIT 3. A large phylogenetic tree of the 3B can be visible for each of the 346 spinach accessions is shown in Supplementary Figure S2–5.

Based on PCA and phylogenetic analysis when PCA = 2 to 10 by GAPIT 3 in the 346 spinach accessions with 8399 randomly selected SNPs distributed on six chromosomes, two sub-populations (clusters) were the most clearly divided in the GWAS panel of the 346 accessions (Fig. S2-1, S2-2, S2-3, S2-4, and Fig. 4). The GWAS panel can also be divided into three subpopulations (clusters or groups) but not for other sub-populations from PCA = 4 to 10 (Fig. 4, Fig. S2-1, S2-2, S2-3). Each of the 346 accessions was arranged into its position in a phylogenetic tree of two sub-populations by the neighbor-joining (NJ) method drawn by GAPIT 3 (Fig. S2-5). The NJ phylogenetic trees of two sub-populations and three sub-populations and the 3D graphical plot of PCA in two sub-populations and three sub-populations were shown in Fig. 4 and listed in Table S1.

Based on 2-cluster, Q1 and Q2 consisted of 301 (87.0%) and 45 (13.0%) accessions, respectively, nevertheless, based on 3-cluster (G1 to G3), there were 301 (87.0%) G1, 26 (7.5%) G2, and 19 (5.5%) G3 accessions, respectively (Table S2). Combining 2- and 3-cluster, all 301 accessions in Q1 stayed at the same cluster G1; but the 45 accessions in Q2 were divided into two groups G2 and G3 with 26 and 19 accessions, respectively (Table S2). The accessions from India, Japan, and Mongolia were grouped into Q2.G2, where the accessions were grouped from cluster Q2 based on two clusters and G2 based on three clusters in the panel; the majority of accessions from China plus all accessions from South Korea and Thailand (but only one accession each of the two countries) to Q2.G3; and the accessions from other countries to Q1.G1 (Table S2).

### Association study

Based on the six models in GAPIT 3 and three models in TASSEL 5 when PCA = 2, 40 SNPs, located on chrs 1, 2, 3, 4, 5, and 6, were associated with the white rust resistance (Table S3). The observed vs expected LOD [−log_10_(p)] distributions in QQ-plots showed a large divergence from the expected distribution (Fig. S3 B), indicating that there were SNPs associated with the white rust resistance in the association panel. The Manhattan plot showed that a dozen SNPs with LOD value greater than 5.42 (significant threshold) across the six GWAS models from GAPIT 3 (Fig. S3 A & C), were associated with white rust resistance.

**Figure 5:**
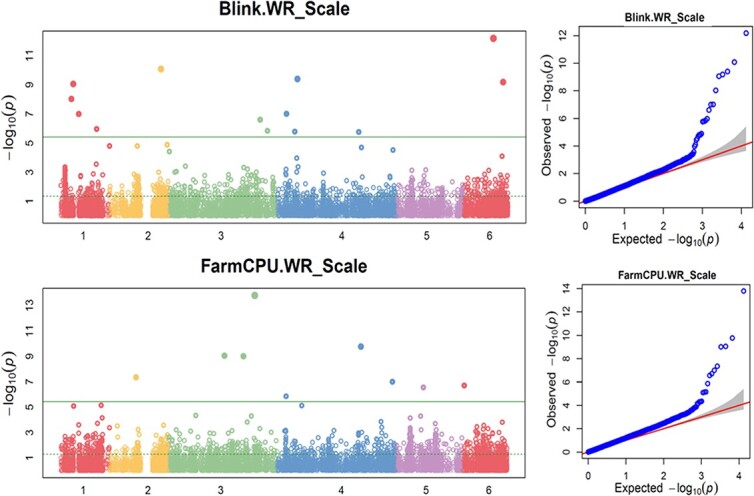
Distribution of Manhattan plot (left) and QQ-plot (right) of GWAS for white rust resistance based on Blink and FarmCPU. For the Manhattan plot, the x-axis presents the spinach 6 chromosomes and the y-axis for the LOD (−log(P-value)) value. For QQ-plot, the x-axis presents LOD (−log(P-value)) value and y-axis for expected LOD (−log(P-value)) value.

**Figure 6:**
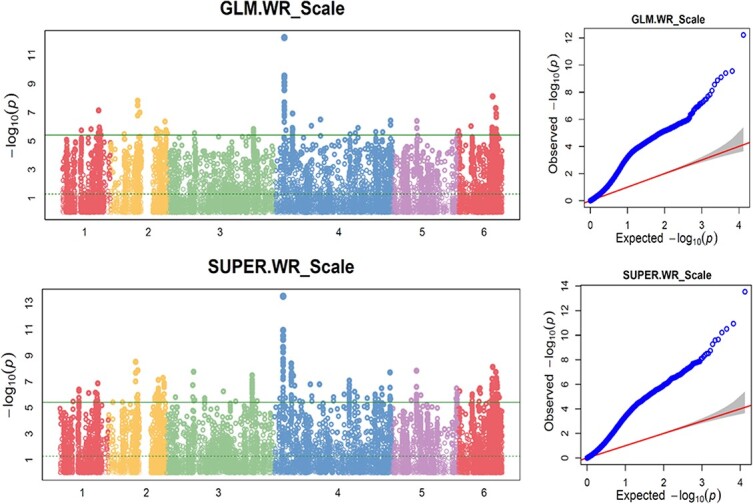
Distribution of Manhattan plot (left) and QQ-plot (right) of GWAS for white rust resistance based on GLM and SUPER. For the Manhattan plot, the x-axis presents the spinach 6 chromosomes and the y-axis for the LOD (−log(P-value)) value. For QQ-plot, the x-axis presents LOD (−log(P-value)) value and y-axis for expected LOD (−log(P-value)) value.

BLINK had SNPs with LOD greater than 5.42 on chrs 1, 2, 3, 4, and 6 and FarmCPU had SNPs with LOD >5.42 on chr 2, 3, 4, 5, and 6 (Fig. 5, Table S3), indicating that there are SNP markers associated with white rust resistance. Gapit.SUPER, Gapit.GLM, Tassel.GLM and Tassel.SMR showed a peak at the region on chr 4, where a dozen SNPs had LOD >5.42 and only this region had SNPs with LOD >10 (Fig. 6, Fig. S4, Table S3), indicating there is a major QTL in the region of chromosome 4 for white rust resistance. However, the Tassel.MLM (Fig. S4), Gapit.MLM, and Gapit.MLMM (Fig. S5) don’t have any SNP with LOD >5.42, but have dozen of SNPs with LOD >3.0 (Fig. S4 and S5) and six SNPs had LOD score > 4.0 or 3.0 in the three models, indicating that there were small-effect QTLs for white rust resistance (Table S3).

**Table 2:** Nine SNP markers associated with white rust resistance in 346 USDA spinach germplasm accessions

SNP^a^	Chr	Position	LOD (−log(P)) value using GAPIT 3^a^						LOD (−Log(P)) value in Tassel^a^			MAF (%)	LOD(−Log(P)) >5.42 in GAPIT3	-LOG(P) > 5.42 in TASSEL 5
			BLINK	FarmCPU	MLM	MLMM	SUPER	GLM	MLM	GLM	SMR			
chr2_53 049 132	2	53 049 132	10.08	2.48	1.32	1.33	5.96	5.61	1.14	4.87	8.65	2.5	Blink,Super,Glm	Smr
chr3_58 479 501	3	58 479 501	2.14	9.05	2.43	2.47	2.46	4.07	1.87	3.08	2.22	38.0	FarmCPU	
chr3_95 114 909	3	95 114 909	6.61	0.55	1.54	1.55	5.52	5.10	1.10	4.10	4.53	3.0	Blink,Super	
chr4_9 176 069	4	9 176 069	7.01	5.86	1.94	1.97	13.54	12.22	1.72	11.82	13.56	3.2	Blink,FarmCPU,Super,Glm	Glm,Smr
chr4_17 807 168	4	17 807 168	0.35	0.81	0.71	0.71	7.38	6.92	0.73	7.89	7.70	2.3	Super,Glm	Glm,Smr
chr4_83 938 338	4	83 938 338	5.76	0.40	1.03	1.04	5.01	5.56	1.19	3.70	3.64	33.8	Blink,Glm	
chr4_87 601 768	4	87 601 768	2.68	3.02	4.02	4.17	2.39	2.50	4.78	2.31	2.18	13.9	MLM + MMLM>4	MLM > 4
chr6_1 877 096	6	1 877 096	1.08	6.71	3.27	3.36	3.33	3.53	3.12	2.55	2.33	8.5	FarmCPU, plus (MLM + MMLM>3)	MLM > 3
chr6_31 287 118	6	31 287 118	12.18	2.98	3.85	3.98	3.64	4.08	3.33	2.97	2.49	20.1	Blink,plus (mlm + mmlm>3)	MLM > 3
SNP	N1	X1	N2	X2	N3	X3	LOD (−log(P)) in *t-*test			A^a^	B^a^	Dominance/recessive for white rust resistance		
							(X1,X2)	(X1,X3)	(X2,X3)					
chr2_53 049 132	329	4.88	0		17	3.56		6.07		T	C	dominant		
chr3_58 479 501	128	4.61	45	4.98	159	4.92	2.30	2.59	0.47	G	T	partial recessive		
chr3_95 114 909	326	4.87	1	4.00	19	3.92		3.79		T	C	complete D		
chr4_9 176 069	325	4.91	1	3.50	20	3.35		8.17		A	G	complete D		
chr4_17 807 168	330	4.88	0		16	3.59		4.41		A	G	dominant		
chr4_83 938 338	45	5.24	157	4.62	73	4.72	8.28	4.16	0.63	G	A	dominant		
chr4_87 601 768	255	4.74	5	5.00	68	5.13	0.52	3.71	0.39	C	A	over-recessive		
chr6_1 877 096	289	4.75	2	5.25	55	5.17	1.61	3.84	0.41	C	A	partial recessive		
chr6_31 287 118	211	4.68	4	5.13	131	5.02	0.81	3.60	0.39	T	C	partial recessive		

a SNP name defined as SNP on the chromosome plus its position on chromosome.

b Lod (-LOG(P)) value, where P is the P value from the six models, BLINK, FarmCPU, MLM, MMLM, SUPER, and GLM in GAPIT 3 R Package, and MLM, GLM, and SMR in TASSEL 5.

a A: Beneficial allele for white rust resistance, B: unbeneficial allele for susceptible

After combining, nine SNPs, chr2_53 049 132, chr3_58 479 501, chr3_95 114 909, chr4_9 176 069, chr4_17 807 168, chr4_83 938 338, chr4_87 601 768, chr6_1 877 096, and chr6_31 287 118, located on chrs 2, 3, 4, and 6, respectively were selected as the associated SNP markers for white rust resistance (Table 2). The SNP, chr2_53 049 132, located at 53049132 bp on chr 2 had a significantly high LOD value at BLINK, SUPER, Gapit.GLM, and Tassel.SMR with LOD values of 10.08, 5.96, 5.61, and 8.65, respectively (>5.42 threshold); high LOD value of 4.87 in Tassel.GLM; but low LOD values of <2.5 in FarmCPU, Gapit.MLM, MLMM, and Tassel.MLM (Table 2) indicates that chr2_53 049 132 was associated with white rust resistance but was not stable across all tested models. SNP, chr3_58 479 501 at 58479501 bp on chr 3 had a large LOD value of 9.05 (>5.42) in FarmCPU, and a LOD value from 2.5 – 5.42 in other eight models except for Tassel.MLM with 1.87 (Table 2), indicating that chr3_58 479 501 was but not strongly associated with white rust resistance. Similar to SNP chr2_53 049 132, SNP, chr3_95 114 909 at 95114909 bp on chr 3 had significant LOD value >5.42 in BLINK and SUPER; a high LOD value (LOD >4.0) at Gapit.GLM, Tassel.GLM; but a low value (LOD <2.0) at FarmCPU, Gapit.MLM, MLMM and Tassel.MLM (Table 2), indicating that chr3_95 114 909 was associated with white rust resistance but was not stable across all tested models. SNP, chr4_9 176 069 at 9176069 bp on chr 4 had a high and significant LOD value >5.42 in BLINK, FarmCPU, SUPER, Gapit.GLM, Tassel.GLM, and SMR, but a low value (LOD <2.0) at Gapit.MLM, MLMM, and Tassel.MLM (Table 2), indicating that chr4_9 176 069 was a comparatively good marker for white rust resistance. The SNP, chr4_17 807 168 at 17807168 bp on chr 4 had a very high and significant LOD value >5.42 in SUPER, Gapit.GLM, Tassel.GLM and SMR but a very low value at other models with LOD <1.0 (Table 2), indicating that chr4_17 807 168 was associated with white rust resistance but did not show stable across all tested models. SNP, chr4_83 938 338 at 83938338 bp on chr 4 had a significant LOD value >5.42 in BLINK and Gapit.GLM; high value (LOD =5.01) at SUPER; and a LOD value >3.5 at both Tassel.GLM and SMR; but a low value (LOD <1.2) at FarmCPU, Gapit.MLM, MLMM, and Tassel.MLM (Table 2), indicating that chr4_83 938 338 was associated with white rust resistance but did not show stability across all tested models. chr4_87 601 768 at 87601768 bp on chr 4 did not have a significant LOD value >5.42 but had value >2.0 across all tested nine models and the highest values across three MLM Models, Gapit.MLM. MLMM, and Tassel.MLM with LOD value >4.0 (Table 2), indicating that the SNP chr4_87 601 768 showed stability across nine models, although the LOD value was not high but significant at P = 0.01. chr6_1 877 096 at 1877096 bp on chr 6 had significant LOD value >5.42 at FarmCPU; high LOD value >3.0 at Gapit.MLM, MLMM, SUPER, Gapit.GLM, and Tassel.MLM; >2.30 at Tassel.GLM and SMR; but low value with 1.08 at BLINK (Table 2), indicating that chr6_1 877 096 was associated with white rust resistance but did not show stability across all tested models. chr6_31 287 118 at 31287118 bp on chr 6 showed the best SNP markers with LOD value >2.4 at all nine models; 12.18 at BLINK; >3.5 at Gapit.MLM, MLMM, SUPER, Gapit.GLM, and Tassel.MLM; >2.9 at FarmCPU and Tassel.GLM; and 2.49 at SMR (Table 2), indicating that the SNP, chr6_31 287 118 was a very stable marker associated with white rust resistance.

### *T-*test for association

*t*-test showed dominance or recessive in each of the selected 40 SNP markers with LOD >2.0 at P = 0.01 level significance for white rust resistance (Table S4). Seven of the 40 SNPs, chr2_50 382 388, chr2_53 049 132, chr3_78126596, chr4_17 691 593, chr4_17 807 168, chr5_25899209, and chr5_51760073, did not have both homozygous genotypes (SNP homozygosity in the panel of 346 spinach accessions) but have heterogeneous genotypes and showed dominance for white rust resistance. In addition, 14 SNPs had only one spinach accession; four SNPs had two accessions; two SNPs had three accessions; and three SNPs had four accessions with homozygosity in one of the SNP alleles, showing dominance or over-dominance (Table S4). Eight SNPs, chr3_58 479 501, chr4_9155049, chr4_9156552, chr4_9163612, chr4_83 938 338, chr4_86 732 255, chr5_28734483, and chr6_41 345 783 showed the significant differences between two homozygous SNP alleles at P = 0.01 level (LOD >2.0) (Table S4). Five of the eight SNPs had only two or three accessions with homozygosity in one of the SNP alleles (Table S4).

Among the nine SNP markers selected (Table S4, Table 2), chr2_53 049 132 showed dominance with allele “T” as a beneficial allele for white rust resistance and “C” as an un-beneficial allele for susceptibility; chr3_95 114 909, chr4_9 176 069, chr4_17 807 168 and chr4_83 938 338 also showed dominance; chr4_87 601 768 showed over-recessive; and chr3_58 479 501, chr6_1 877 096, and chr6_31 287 118 showed partial-recessive.

### Candidate genes for white rust resistance

A total of 121 genes were listed in Supplementary Table S5 and they were located within 50 Kb distance from the 40 associated SNP markers in Table S3. All Leucine-Rich Repeat (LRR) genes plus those with less than 1 Kb distance from the associated SNP markers were listed in Table 3, where 13 genes were located at 12 associated SNP regions. Six SNPs were inside six genes and three SNPs were with a distance less than 1 Kb from a gene, respectively. Six SNPs were located less than 50 Kb from five disease resistance gene analogues encoding LRR domains (Table 3).

**Table 3:** List of 13 genes located at 12 associated SNP regions including 6 SNPs on the 6 genes and 3 SNPs with a distance less than 1 Kb with a gene, respectively, and 6 SNPs with a distance less than 50 Kb with 5 disease resistance gene analogue LRR domain

Gene ID	Chr	Gene Start	Gene End	Gene Description	Associated SNP	Chr	SNP Position	Distance from the start position of the gene	Distance from the end position of the gene	Distance (Kb)
Spo15817	2	28 461 449	28 471 213	ABH1	chr2_28 471 392	2	28 471 392	9943	179	<1 kb
Spo01590	2	50 372 467	50 388 526	Quinoprotein amine dehydrogenase, beta chain-like / RIC1-like guanyl-nucleotide exchange factor	chr2_50 382 388	2	50 382 388	9921	−6138	on gene
Spo01686	2	50 399 687	50 401 105	Receptor-like kinase, Leucine-rich repeat (LRR)				−17 299	−18 717	<20Kb
Spo23694	2	53 041 260	53 050 511	Serine decarboxylase family protein	chr2_53 049 132	2	53 049 132	7872	−1379	on gene
Spo12068	4	9 097 039	9 101 229	Receptor-like protein kinase, Leucine-rich repeat (LRR)	chr4_9 077 455	4	9 077 455	−19 584	−23 774	<30Kb
Spo12071	4	9 151 132	9 154 780	60S ribosomal protein L7a, putative	chr4_9 152 378	4	9 152 378	1246	−2402	on gene
Spo12072	4	9 170 439	9 178 052	Lecithin:cholesterol acyltransferase family protein	chr4_9 170 963	4	9 170 963	524	−7089	on gene
Spo20901	4	17 644 108	17 646 417	Leucine-rich repeat (LRR) receptor-like protein kinase	chr4_17 691 593	4	17 691 593	47 485	45 176	<50Kb
Spo20900	4	17 674 521	17 684 091	Receptor-like kinase, Leucine-rich repeat (LRR)				17 072	7502	<10Kb
Spo14612	4	20 529 360	20 535 771	Calmodulin binding protein	chr4_20 532 790	4	20 532 790	3430	−2981	on gene
Spo08236	4	86 730 376	86 739 358	Nuclear transport factor 2B	chr4_86 732 255	4	86 732 255	1879	−7103	on gene
Spo04510	6	31 326 235	31 330 354	Receptor-like protein kinase 2, Leucine-rich repeat (LRR)	chr6_31 287 112	6	31 287 112	−39 123	−43 242	<40Kb
					chr6_31 287 118	6	31 287 118	−39 117	−43 236	<40Kb
Spo25888	6	41 342 127	41 345 503	Dihydroflavonol 4-reductase	chr6_41 345 783	6	41 345 783	3656	280	<1 kb

The six gene models, Spo01590, Spo23694, Spo12071, Spo12072, Spo14612, and Spo08236 contain a SNP marker, chr2_50 382 388, chr2_53 049 132, chr4_9 152 378, chr4_9 170 963, chr4_20 532 790, and chr4_86 732 255 respectively, on chrs 2 and 4 (Table 3). Whether these six gene models are related to white rust resistance needs further study. The Leucine-Rich Repeat (LRR) gene model, Spo01686 located at 50399687 – 50401105 bp on chr 2, is based on Sp75 spinach genome reference located near SNP marker chr2_50 382 388 (distance of 17.299 Kbp). Spo12068, located at 9097039 – 9101229 bp on chr 4, is located near SNP marker chr4_9 077 455 (distance of 19 548 Kbp). Both LRR models, Spo20901 and Spo20900, located at 17644108 -17646417 bp and 17 674 521 – 17 684 091 bp on chr 4, located near the SNP marker chr4_17 691 593 with distance 45.176 Kbp and 7.502 Kbp, respectively. Spo04510, located at 31326235 – 31330354 bp in chr 6 is near the SNP marker chr6_31 287 112 (39.123,kbp) (Table 3). Further studies are needed to evaluate whether the five LRR genes are related to white rust resistance.

### Genomic prediction of white rust resistance

#### Genomic prediction with different ratios of the training set to testing set

In this study, there were nine ratios between training and testing sets, two GP models, and three SNP sets making a total of 54 combinations. The average r-value (r_${\bar{Y}} $100_) and its standard error (SE) from the 100 runs for each combination are listed in Table S6 and the 54 averaged r values (r_${\bar{Y}} $100_) displayed in charts created by R package divided by two models: left half from BL (Bayesian LASSO) and right half from rrBLUP, and grouped by the nine folds with three SNP sets (Fig. S6).

The nine-fold sets had similar, although not identical, averaged r values (r_${\bar{Y}} $100_) in each SNP set using the same model, either BL or rrBLUP (Table S6, Fig. S6). From rrBLUP, the r-value averaged 0.67, ranged from 0.63 (2 fold) to 0.69 (8- or 9- fold) in all.13235-SNP; averaged 0.39 and ranged from 0.38 to 0.40 in 40-SNP set; and averaged 0.23 and ranged from 0.22 to 0.25 in the 9-SNP marker set. From BL, the r-value averaged 0.82 and ranged from 0.76 to 0.84 in all.13235-SNP set; averaged 0.73 and ranged from 0.72 to 0.74 in the 40-SNP marker set; average 0.59 and ranged from 0.58 to 0.60 in 9-SNP.marker set (Table S6). Overall, the 2-fold had a low r-value but had a smaller SE. However, the SE increased when increasing the fold number. In general, BL model had higher r-value than rrBLUP. The all.13235.SNP set had higher value than the other two sets and the 40-SNP.marker set had a higher r-value than the 9-SNP.marker set (Table S6, Fig. S6).

#### Genomic prediction with different SNP numbers

GP was performed with eight different SNP number sets (9, 40, 100, 200, 500, 1000, 2000, and 4836 SNPs) selected from three different SNP groups in cross-predictions for white rust resistance using BL model in three SNP sets: Set.13235SNP, Set.4836SNP.select, and Set.8839SNP.random (Datasets available at FigShare: https://doi.org/10.6084/m9.figshare.17283194). There were 24 combinations for GP analysis, consisting of eight SNP number sets selected from three SNP groups. Each GP analysis was run for 100 times to calculate GP statistical parameters and r values. The average r-value (r_${\bar{Y}} $100_) and SE of 100 runs for each GP combination are presented in the Table S7 and Fig. S7.

The results showed that the average r value (r_${\bar{Y}} $100_) decreased when decreasing the SNP number (Table S7 and Fig. S7). From the Set.13235SNP, the average r-value (r_${\bar{Y}} $100_) was 0.79 when 4836 SNPs were used and decreased to 0.25 when 9 SNPs were used. In the Set.4836SNP.select, the average r-value (r_${\bar{Y}} $100_) was 0.82 when 4836 SNPs were used and decreased to 0.31 when 9 SNPs were used. In the two SNP groups, the r-value was higher than 0.50 when 100 SNPs or more were used. But the average r-value (r_${\bar{Y}} $100_) was very low in the Set.8839SNP.random of the SNP group; ranged from 0.19 to 0.07; and the highest was only 0.19 when 4836 SNPs were used (Table S7, Fig. S7), indicating that random SNP group without associated markers included can’t be used for white rust resistance in GS, but using associated SNP marker will increase the selection efficiency; and when > = 100 SNPs with associated markers can be used as a set to select white rust resistance in GS.

#### Genomic prediction using different models

**Figure 7:**
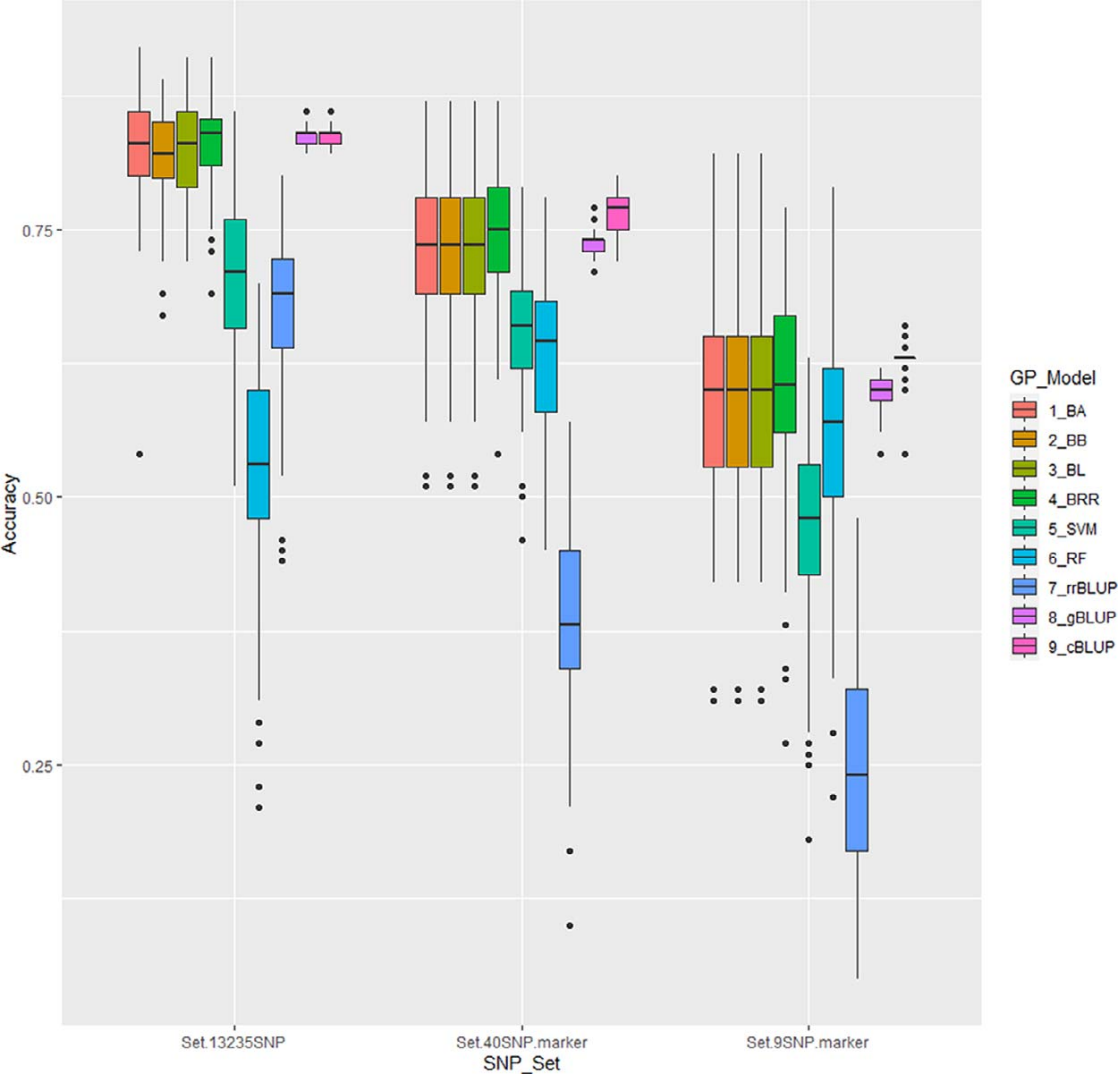
Genomic prediction (r-value) of nine GP models, BA, BB, BL, BRR, SVM, RF, rrBLUP, gBLUP, and cBLUP for white rust resistance among three SNP sets in cross-prediction for white rust resistance in 346 spinach accessions for three SNP sets, all 13 235 SNPs (left group), 40 SNP markers (middle group) and 9 SNP markers (right group).

Nine GP models (BA, BB, BL, BRR, SVM, RF, rrBLUP, gBLUP, and cBLUP) were used to conduct GP for white rust resistance among seven SNP sets: all, 40 m, 9 m, 40r, 9r, 40rr, and 9rr, where all signifies all 13 235 SNP set; 40 m is the 40 SNP markers in the Table S3; 9 m is the 9 SNP markers listed in Table 2; 40r is a SNP set consisted of 40 SNPs randomly selected from the 13 235 SNP set; 9r is a SNP set of 9 SNPs randomly selected from the 13 235 SNP set; 40rr is a set consisting of 40 SNPs randomly selected from the random 8839 SNP set; and 9r is a set of 9 SNPs randomly selected from the random 8839 SNP set (Table S8, Fig. 7). The six GP models (BA, BB, BL, BRR, gBLUP, and cBLUP) had similar high average r-value (r_${\bar{Y}} $100_): > = 0.82 in all.13235-SNP set; > = 0.73 in the 40-SNP marker set; and > =0.59 in the 9-SNP marker set. The other three models, SVM, RF, and rrBLUP, still had a high average r-value (r_${\bar{Y}} $100_) > =5.2 in the all.

SNP set. SVM and RF have r-value > = 0.63 in the 40-SNP marker, and > =0.47 in 9-SNP marker set, but rrBLUP had a low value with 0.38 and 0.25, respectively (Table S8, Fig. 7), indicating that the six GP models (BA, BB, BL, BRR, gBLUP, and cBLUP) are good GP models to be utilized in GS for selecting white rust resistance in spinach. All nine models had low r_${\bar{Y}} $100_ values in the four random sets (40r, 9r, 40rr, and 9rr), suggesting that we can’t use a small SNP number randomly selected from a million SNP set in GS for white rust resistance.

#### Genomic prediction using GWAS-derived SNP markers

A higher r-value (r_${\bar{Y}} $100_) were observed when using the GWAS-derived SNP marker sets: > = 0.73 in 40-SNP marker set (m40) and > =0.59 in 9-SNP marker set (9 m) across the six GP models (BA, BB, BL, BRR, gBLUP, and cBLUP) (Table S8, Fig. 7). An averaged 0.75 of r_${\bar{Y}} $100_ value in 40 m set and 0.61 in 9 m set were calculated across nine GP models, which were much higher than those from the four randomly selected SNP sets: either 40r, 9r, 40rr, or 9rr across all tested nine GP models (Table S8, Fig. 7), indicating that the GWAS-derived SNP marker sets had high PA and suggesting that we can use the GWAS-derived SNP markers in GS for selecting white rust resistance.

## Discussion

### Evaluation of white rust

White rust is a non-culturable oomycete pathogen making it difficult to screen and select spinach genotypes for resistance. Currently, no efficient method has been developed to evaluate white rust resistance in greenhouse or growth chamber conditions as disease severity among known resistant and susceptible genotypes is difficult to discriminate in a single disease cycle [70]. Therefore, field evaluations, whereby spinach genotypes can be evaluated over multiple secondary infection cycles over a longer period of time, can be used to evaluate and select white rust-resistant lines in spinach [4]. For example, some spinach genotypes that are known to be highly resistant to white rust get infected, but the lesions develop somewhat slower and the rust pustules may not even break through the epidermis. As a result, fewer infections occur on a given plant and the difference between a highly susceptible and highly resistant line in a single infection cycle may not be noticeable, but the differences become more pronounced over a longer period of time under field conditions. However, even in a white rust disease nursery, as was used in this study, disease severity is still highly dependent on the environment from year to year, resulting in a low and unpredictable selection efficiency. Establishing a uniform spread of consistent white rust nurseries under field setting is difficult to accomplish, requiring multiple years of no-rotation spinach crop to build enough inoculum in the soil. In this study, the 346 USDA spinach germplasm accessions were evaluated for white rust resistance in the Del Monte White Rust Nursery in Crystal City, Texas, for three years during the three winter seasons of 2015–16, 2016–17, and 2017–18. The nursery was used for spinach white rust evaluation for commercial spinach hybrids, germplasm, and breeding lines where heavy disease pressure had consistently been observed for 30 years when environmental conditions were favorable for the disease. Because the disease severity was relatively low in the winter seasons 2015–2016 and 2016–2017 including the known susceptible control lines, the white rust disease severity ratings were not robust (i.e. false low ratings due to escape from disease). However, disease severity was high in the winter 2017–2018 season due to the favorable environment. Therefore, this 2017–2018 disease severity report was only used to conduct GWAS and identify SNP markers associated with white rust. Based on the white rust phenotypic data, 23 of 346 spinach accessions showed relatively high resistance to white rust. The accessions showing higher resistance with a disease severity scale of 2.0 or less (Table 1 & S1) can be used as parents in breeding programs to develop white rust-resistant lines and cultivars.

### Genome-wide association study and SNP marker identification for white rust resistance

In this study, GWAS was performed in two steps. In the first step, 4836 SNP markers associated with white rust resistance were identified from 2 357 260 SNPs using TASSEL 5. In the second step, 13 235 SNPs (the 4836 SNPs from the first step plus the randomly selected 8399 SNPs) were used to conduct GWAS by implementing multiple models, including six models in GAPIT 3, three models in TASSEL 5, and *t*-test. Forty SNP markers were associated with white rust resistance with LOD >5.42 in one of the six tested MLM models (Gapit.MLM, MLMM, SUPER, FarmCPU, BLINK, or Tassel.MLM). After combining analysis of the six models in GAPIT 3 and three models in TASSEL 5, nine SNPs located on chrs 2, 3, 4, and 6 were relatively consistent across the models and were selected as the associated SNP markers for white rust resistance in this study (Table 2). Awika et al. (2019) [30] conducted GWAS analysis for white rust resistance in a panel of 267 spinach accessions with 6111 SNPs and reported a total of 448 minor alleles (SNPs) associated with white rust severity. None of the 448 SNPs reported by Awika et al. (2019) [30] was validated in this study since their approach targeted factors associated with susceptibility. Therefore, it is possible to combine resistance and susceptible associated SNPs found in this study to improve spinach resistance. As expected, we observed differences in the number of identified associated SNPs when using TASSEL 5 vs GAPIT 3 GWAS tools or different models such as BLINK, FarmCPU, GAPIT.MLM and GAPIT.GLM. The same differences have been widely reported and discussed in several publications [30–36, 53]. In this study, we selected SNPs as the markers associated with white rust resistance by multiple models combined, including six models in GAPIT 3 and three models in TASSEL 5 if the SNP had a significant LOD value across multiple models.

### Candidate genes for white rust resistance

In this study, a total of 121 genes were identified to be located within 50 Kb distance from the 40 associated SNP markers (Table S3). Thirteen genes located at 12 associated SNP regions were selected as condidates for white rust resistance, among them, five were disease resistance gene analogue with LRR domain (Table 3). However, further studies are needed to confirm whether the five LRR genes are related to white rust resistance.

Despite the success of GWAS in identifying genetic loci associated with several agronomic and disease resistance-related traits, it will be challenging to pinpoint the causal gene in each of these loci. A successful GWAS only identifies probable genomic regions but requires subsequent characterization for validating the actual identification of causal relationship with disease using proteomics/ transcriptional profiling. Given the complexity and nature of the white rust, most genes identified in our study, whether a given gene is likely to be involved in determining a resistant phenotype alone would need cloning individual genes in the appropriate genetic background. Most verified plant disease resistance genes isolated to date contain a nucleotide-binding site and leucine-rich repeat (NBS-LRR) domains- similar to the one we identified in this study. Activated NB-LRRs represent a tip of the signaling cascade that triggers defense responses and not the causal genes defining the resistance alone. It will be impulsive to correlate expression patterns of the identified candidate genes and disease reaction. The percent variation (R-square%) explained by individual SNP (Table 3) shows the strength of these variants at the evolutionary and population levels in defining resistance. Hence, the genes identified in this study open new avenues to design white rust resistance through systematic integration of selected accessions in the breeding program of spinach and other closely related species. Detailed characterization of these genes, although intriguing, is beyond the scope of this paper. However, we will continue pursuing the relationship of these genes in resistance mechanisms as a future work through additional studies.

### Genomic prediction

In this study, the nine-fold sets from 2 fold (1:1) to 10-fold (9:1) had similar, although not identical, averaged r values (r_${\bar{Y}} $100_) in each SNP set using the same model, either BL or rrBLUP (Table S6, Fig. S6). When increasing the fold number, the SE was also increased, suggesting that a larger training set and the smaller testing set would increase the error. The 2-fold set has a smaller r-value and showed that a smaller training set would have less PA with a smaller r-value. Shi et al. (2021) [53] also reported that different training/testing ratio sets showed similar trends in GP analysis for soybean cyst nematode resistance in common bean. Ravelombola et al. (2021) [49] reported similar results for growth habit, flowering time, and grain yield in the cowpea population evaluated under drought conditions. Keller et al. (2020) [71] reported that a training set of <30% reduces PA due to a small sized training set that results in overfitting of the model. They also noted that increasing training set >80% leads to large variation between cross-validations with a small validation set.

The six GP models (BA, BB, BL, BRR, gBLUP, and cBLUP) had similar high average r-value (r_${\bar{Y}} $100_): > = 0.82 in all.13235-SNP set, > = 0.73 in the 40-SNP marker set, and > =0.59 in the 9-SNP marker set. The three models, SVM, RF, and rrBLUP, still had high average r-value (r_${\bar{Y}} $100_) > =5.2 in the all SNP set, > = 0.63 in the 40-SNP marker, and > =0.47 in 9-SNP marker set, but rrBLUP had low value with 0.38 and 0.25, respectively (Table S8, Fig. 7), indicating that the six GP models are good GP models to be utilized in GS for selecting white rust resistance in spinach. Usually, the Bayesian models such as BA, BB, BL, and BRR had high PA with higher r-value [53]. The gBLUP and cBULP models in GAPIT tool had a lower r-value than other models for SCN resistance in common bean [53]. Since 2021, the gBLUP and cBLUP models running in GAPIT 3 tool in Zhiwu Zheng’s lab has improved their prediction and created higher PA than other models as shown in this study and the two models had the highest average r-value (r_${\bar{Y}} $100_) with the smallest SE (Table S8, Fig. 7), indicating their efficiency in GS for white rust resistance in spinach.

GP was thirdly performed with eight different SNP number sets from 9 to 4836 SNPs for white rust resistance using BL model. The results showed that the average r-value (r_${\bar{Y}} $100_) decreased when decreasing the SNP number (Table S7 and Fig. S7). From the Set.13235SNP, the average r-value (r_${\bar{Y}} $100_) was 0.79 when 4836 SNPs were used and decreased to 0.25 when 9 SNPs were used. In the Set.4836SNP.select, the average r-value (r_${\bar{Y}} $100_) was 0.82 when 4836 SNPs were used and decreased to 0.31 when 9 SNPs were used. In the two SNP groups, the r-value was higher than 0.50 when 100 SNPs or more were used. However, the average r-value (r_${\bar{Y}} $100_) was very low in randomly selected set: Set.8839SNP.random, which was randomly selected from 217 531 SNPs (4.06%); ranged from 0.07 to 0.19 (Table S7, Fig. S7), indicating that random SNP group without associated markers included cannot be used for white rust resistance in GS, but using associated SNP marker will increase the selection efficiency. From this study, 100 or more SNPs with associated markers can be used as a set to select white rust resistance in GS. In most reports, the smaller the number of SNPs resulted in lowering the PA [40, 53, 71, 72]. Zhang et al. (2016) [40] estimated PA (r-value) of seed size of 309 soybean accessions and reported r = 0.85 using 2000 SNPs or 31 045 SNPs; and lowered to 0.8 when 1000 SNPs or 500 SNPs were used.

In this study, GP was performed using GWAS-derived SNP markers, either 4836 selected SNPs, 40-SNP marker set, or 9-SNP marker set had higher PA (r-value) than the randomly selected SNP sets in all of the tested GP models (Table S6 and S8). The results suggest the advantage of using the GWAS-derived SNP markers in GS for white rust resistance. Zhang et al. (2016) [40] reported that the r values were 25% higher when using GWAS-derived SNP markers than using the same number of randomly selected SNPs for seed size in soybean. Qin et al. (2019) [72] also reported that the average r values were higher when using SNP markers for 15 amino acid contents in soybean seeds. Spindel et al. (2016) [73] developed a GS model that combines RR-BLUP with GWAS derived-markers and reported that this new model outperformed for a variety of traits in multiple environments. Thus, using GWAS-derived SNP markers to perform GS is an approach that combines MAS and GS and can be used in the real-world breeding programs. However, the predictive ability may be biased when GWAS-associated SNP markers are used to predict the GEBVs in the same GWAS panel. The GP will probably be lower if prediction performance is tested in other panels with different individuals. Similar approaches have been tested for many traits in several crops and found it practical to do genome breeding using GWAS-derived SNP markers [49–51, 53, 72]. Therefore, GP approach combining both MAS and GS through GEBVs using associated SNP markers would be valuable in molecular breeding for white rust resistance in spinach and for other quantitative traits in other plant species, and assessment of genomic prediction potential is ongoing on several important traits in spinach [74].

## Conclusion

In this study, 346 USDA spinach germplasm accessions were phenotyped for white rust resistance under field conditions; 23 accessions showed white rust resistance or intermediate resistance with a disease rate 3.0 or less based on 0–10 scale; and the seven accessions, NSL 6098, PI 175311, PI 220686, PI 224959, PI 226671, PI 227045, and PI 648958 showed the highest white rust resistant levels with a score of 2.0 or less, indicating their suitability as parents in breeding programs to develop white rust-resistant hybrids and cultivars. Genome-wide association study (GWAS) was performed in the 346 accessions with 13 235 SNPs and identified nine SNPs, chr2_53 049 132, chr3_58 479 501, chr3_95 114 909, chr4_9 176 069, chr4_17 807 168, chr4_83 938 338, chr4_87 601 768, chr6_1 877 096, and chr6_31 287 118, located on chromosomes 2, 3, 4, and 6 associated with white rust resistance. Genomic prediction (GP) was tested for prediction accuracy (PA) using Pearson’s correlation coefficient (r) between the genomic estimation breeding value (GEBV) and the observed values. High averaged r values were observed in each SNP set using different GP models and up to 0.84 when using GWAS-derived significant SNP markers. The SNP markers and the high PA can provide valuable information for breeders to improve spinach by marker-assisted selection (MAS) and genomic selection (GS).

## Supplementary Material

Web_Material_uhac069

## Data Availability

The datasets presented in this study are available in Tables, Figures, Supplementary Tables, and Supplementary Figures. The SNP data are available in FigShare https://doi.org/10.6084/m9.figshare.17283194. The accession number(s) used in this study can be found in the article/Supplementary Material.

## References

[1] Davis W , Lucier G. Vegetable and pulses outlook No. (VGS-366) 68 pp: April 2021. United States Dep Agric Econ Res Serv. 2021.

[2] Dicoteau DR . Vegetable Crops. New Jersey: Printice Hall; 2000.

[3] Morelock, T. E. & Correll, J. C. Spinach. In: J. Prohens and F. Nuez (eds.), Handbook of Plant Breeding, Vegetables I, Asteraceae, Brassicaceae, Chenopodicaceae, and Cucurbitaceae. New York: Springer, 2008, 189–218.

[4] Brandenberger LP , Correll JC, Morelock TE et al. Characterization of resistance of spinach to white rust (*Albugo occidentalis*) and downy mildew (*Peronospora farinosa* f.sp. *spinaciae*). Phytopathology. 1994;84:431–7.

[5] Correll JC , Bluhm BH, Feng C et al. Spinach: better management of downy mildew and white rust through genomics. Eur J Plant Pathol. 2011;129:193–205.

[6] Choi D , Priest MJ. A key to the genus Albugo. Mycotaxon. 1995;53:261–72.

[7] Correll JC , Black MC, Koike ST et al. Economically important diseases of spinach. Plant Dis. 1994;78:653–60.

[8] Goreta S , Leskovar DI. Screening spinach cultivars for white rust resistance and bolting. HortTechnology. 2006;16:162–6.

[9] Vakalounakis DJ , Doulis AG. First record of white rust, caused by *Albugo occidentalis*, on spinach in Greece. Plant Dis. 2013;97:1253. 10.1094/PDIS-02-13-0198-PDN 30722444

[10] Correll JC , Feng CD, Liu B. First report of white rust (*Albugo occidentalis*) of spinach in Mexico. Plant Dis. 2017;101:511.

[11] Soylu S , Kara M, Kurt S et al. First report of white blister rust disease caused by Albugo occidentalis on spinach in Turkey. Plant Dis. 2018;102:826.

[12] Black MC , Dainello FJ, Kunkel TE. Fungicide evaluations for spinach white rust control on resistant and susceptible cultivars. Fungic Nematic Tests. 1992;47:1128.

[13] Dainello F , Black M, Disease TKP et al. Control of white rust of spinach with partial resistance and multiple soil applications of metalaxyl granules. Plant Dis. 1990;74:913–6.

[14] Dainello F , Heineman R. Relative white rust resistance and adaptability of spinach varieties in Southwest Texas. Texas Agricultural Experiment Station. 1981;3878:7 pages.

[15] Dohm JC , Minoche AE, Holtgrawe D et al. The genome of the recently domesticated crop plant sugar beet (*Beta vulgaris*). Nature. 2014;505:546–9. 24352233 10.1038/nature12817

[16] Hirakawa H , Toyoda A, Itoh T et al. A spinach genome assembly with remarkable completeness, and its use for rapid identification of candidate genes for agronomic traits. DNA Res. 2021;28. 10.1093/dnares/dsab004 PMC8231376 34142133

[17] Hulse-Kemp AM , Bostan H, Chen S et al. An anchored chromosome-scale genome assembly of spinach improves annotation and reveals extensive gene rearrangements in euasterids. Plant Genome. 2021;14:e20101. 34109759 10.1002/tpg2.20101 PMC12806983

[18] Xu C , Jiao C, Sun H et al. Draft genome of spinach and transcriptome diversity of 120 Spinacia accessions. Nat Commun. 2017;8:15275. 28537264 10.1038/ncomms15275 PMC5458060

[19] Cai X , Sun X, Xu C et al. Reference genome and resequencing of 305 accessions provide insights into spinach evolution, domestication and genetic basis of agronomic traits. Nat Commun. 2021;12:7246. 34903739 10.1038/s41467-021-27432-z PMC8668906

[20] Davey JW , Hohenlohe PA, Etter PD et al. Genome-wide genetic marker discovery and genotyping using next-generation sequencing. Nat Rev Genet. 2011;12:499–510. 21681211 10.1038/nrg3012

[21] Li H , Peng Z, Yang X et al. Genome-wide association study dissects the genetic architecture of oil biosynthesis in maize kernels. Nat Genet. 2013;45:43–50. 23242369 10.1038/ng.2484

[22] Yano K , Yamamoto E, Aya K et al. Genome-wide association study using whole-genome sequencing rapidly identifies new genes influencing agronomic traits in rice. Nat Genet. 2016;48:927–34. 27322545 10.1038/ng.3596

[23] Ma J , Shi A, Mou B et al. Association mapping of leaf traits in spinach (*Spinacia oleracea* L.). Plant Breed. 2016;135:399–404.

[24] Chitwood J , Shi A, Mou B et al. Population structure and association analysis of bolting, plant height, and leaf erectness in spinach. HortScience. 2016;51:481–6.

[25] Shi A , Mou B, Cheng ZM. Genetic diversity and association analysis of leafminer (*Liriomyza langei*) resistance in spinach (*Spinacia oleracea*). Genome. 2016;59:581–8. 27490441 10.1139/gen-2016-0075

[26] Shi A , Mou B, Correll JC. Association analysis for oxalate concentration in spinach. Euphytica. 2016;212:17–28.

[27] Shi A , Mou B, Correll J et al. SNP association analysis of resistance to Verticillium wilt (*Verticillium dahliae* Kleb.) in spinach. Aust J Crop Sci. 2016;10:1188–96.

[28] Shi A , Mou B, Correll J et al. Association analysis and identification of SNP markers for Stemphylium leaf spot (*Stemphylium botryosum* f. sp. *spinacia*) resistance in spinach (*Spinacia oleracea*). Am J Plant Sci. 2016;07:1600–11.

[29] Qin J , Shi A, Mou B et al. Genetic diversity and association mapping of mineral element concentrations in spinach leaves. BMC Genomics. 2017;18:941. 29202697 10.1186/s12864-017-4297-y PMC5715654

[30] Awika HO , Marconi TG, Bedre R et al. Minor alleles are associated with white rust (*Albugo occidentalis*) susceptibility in spinach (*Spinacia oleracea*). Hortic Res. 2019;6:129. 31814982 10.1038/s41438-019-0214-7 PMC6885047

[31] Awika HO , Bedre R, Yeom J et al. Developing growth-associated molecular markers via high-throughput phenotyping in spinach. Plant Genome. 2019;139:402–18. 10.3835/plantgenome2019.03.0027 PMC12810063 33016585

[32] Awika HO , Cochran K, Joshi V et al. Single-marker and haplotype-based association analysis of anthracnose (*Colletotrichum dematium*) resistance in spinach (*Spinacia oleracea*). Plant Breed. 2020;139:402–18.

[33] Bhattarai G . Genetic resistance to the downy mildew pathogen and mapping the RPF resistance loci in spinach. In: Doctoral Dissertation. University of Arkansas: Fayetteville, USA, 2019. Available at https://scholarworks.uark.edu/etd/3442.

[34] Bhattarai G , Shi A, Feng C et al. Genome wide association studies in multiple spinach breeding populations refine downy mildew race 13 resistance genes. Front Plant Sci. 2020;11. 10.3389/fpls.2020.563187 PMC7609621 33193490

[35] Bhattarai G , Yang W, Shi A et al. High resolution mapping and candidate gene identification of downy mildew race 16 resistance in spinach. BMC Genomics. 2021;22:478. 34174825 10.1186/s12864-021-07788-8 PMC8234665

[36] Olaoye D . Resistance Screening and Association Mapping for Resistance to the Downy Mildew Pathogen of Spinach. In: MS Thesis. University of Arkansas: Fayetteville, USA, 2021. Available at https://scholarworks.uark.edu/etd/4107.

[37] Brandenberger LP , Correll JC, Morelock TE. Identification of and cultivar reactions to a new race (race 4) of *Peronospora farinosa* f. sp. *spinaciae* on spinach in the United States. Plant Dis. 1991;75:630–4.

[38] Albrecht T et al. Genome-based prediction of testcross values in maize. Theor Appl Genet. 2011;123:339–50. 21505832 10.1007/s00122-011-1587-7

[39] Bernardo R , Yu J. Prospects for genome-wide selection for quantitative traits in maize. Crop Sci. 2007;47:1082–90.

[40] Zhang J , Song Q, Cregan P Jiang G-L. Genome-wide association study, genomic prediction and marker-assisted selection for seed weight in soybean (Glycinemax). Theor Appl Genet. 2016;129 (1):117–30. 26518570 10.1007/s00122-015-2614-x PMC4703630

[41] Heffner EL , Jannink JL, Iwata H et al. Genomic selection accuracy for grain quality traits in biparental wheat populations. Crop Sci. 2011;51:2597–606.

[42] Heffner EL , Sorrells ME, Jannink JL. Genomic selection for crop improvement. Crop Sci. 2009;49:1–12.

[43] Onogi A et al. Toward integration of genomic selection with crop modelling: the development of an integrated approach to predicting rice heading dates. Theor Appl Genet. 2016;129:805–17. 26791836 10.1007/s00122-016-2667-5

[44] Poland J , Endelman J, Dawson J et al. Genomic selection in wheat breeding using genotyping-by-sequencing. Plant Genome. 2012;5.

[45] Poudel HP , Sanciangco MD, Kaeppler SM et al. Genomic prediction for winter survival of lowland switchgrass in the northern USA. *G3 genes*. Genomes, Genet. 2019;9:1921–31. 10.1534/g3.119.400094 PMC6553536 30971392

[46] Würschum T , Reif JC, Kraft T et al. Genomic selection in sugar beet breeding populations. BMC Genet. 2013;14:85–8. 24047500 10.1186/1471-2156-14-85 PMC3848454

[47] Hernandez CO , Wyatt LE, Mazourek MR. Genomic prediction and selection for fruit traits in winter squash. G3 Genes|Genomes|Genetics. 2020;10:3601–10. 32816923 10.1534/g3.120.401215 PMC7534422

[48] Xavier A , Muir WM, Rainey KM et al. Assessing predictive properties of genome-wide selection in soybeans. G3 (Bethesda). 2016;6:2611–6. 27317786 10.1534/g3.116.032268 PMC4978914

[49] Ravelombola W , Shi A, Huynh BL. Loci discovery, network-guided approach, and genomic prediction for drought tolerance index in a multi-parent advanced generation intercross (MAGIC) cowpea population. Hortic Res. 2021;8:24. 33518704 10.1038/s41438-021-00462-w PMC7848001

[50] Ravelombola WS , Qin J, Shi A et al. Genome-wide association study and genomic selection for tolerance of soybean biomass to soybean cyst nematode infestation. PLoS One. 2020;15:e0235089. 32673346 10.1371/journal.pone.0235089 PMC7365597

[51] Ravelombola WS , Qin J, Shi A et al. Genome-wide association study and genomic selection for soybean chlorophyll content associated with soybean cyst nematode tolerance. BMC Genomics. 2019;20. 10.1186/s12864-019-6275-z PMC6882315 31775625

[52] Bao Y , Vuong T, Meinhardt C et al. Potential of association mapping and genomic selection to explore PI 88788 derived soybean cyst nematode resistance. Crop science. 2014;7.

[53] Shi A , Gepts P, Song Q et al. Genome-wide association study and genomic prediction for soybean cyst nematode resistance in USDA common bean (Phaseolus vulgaris) Core collection. Front Plant Sci. 2021;12:624156. 34163495 10.3389/fpls.2021.624156 PMC8215670

[54] Wang J , Zhang Z. GAPIT version 3: boosting power and accuracy for genomic association and prediction. Genomics Proteomics Bioinformatics. 2021;19:629–40. 34492338 10.1016/j.gpb.2021.08.005 PMC9121400

[55] Zeng J , Pszczola M, Wolc A et al. Genomic breeding value prediction and QTL mapping of QTLMAS2011 data using Bayesian and GBLUP methods. BMC Proc. 2012;6:S7. 22640755 10.1186/1753-6561-6-S2-S7 PMC3363161

[56] Van Dijk EL , Jaszczyszyn Y, Thermes C. Library preparation methods for next-generation sequencing: tone down the bias. Exp Cell Res. 2014;322:12–20. 24440557 10.1016/j.yexcr.2014.01.008

[57] Collins K , Zhao K, Jiao C et al. SpinachBase: a central portal for spinach genomics. Database (Oxford). 2019;2019:baz072. 31211398 10.1093/database/baz072 PMC6580994

[58] Li H , Durbin R. Fast and accurate short read alignment with burrows-wheeler transform. Bioinformatics. 2009;25:1754–60. 19451168 10.1093/bioinformatics/btp324 PMC2705234

[59] McKenna A , Hanna M, Banks E et al. The genome analysis toolkit: a MapReduce framework for analyzing next-generation DNA sequencing data. Genome Res. 2010;20:1297–303. 20644199 10.1101/gr.107524.110 PMC2928508

[60] Lipka AE , Tian F, Wang Q et al. GAPIT: genome association and prediction integrated tool. Bioinformatics. 2012;28:2397–9. 22796960 10.1093/bioinformatics/bts444

[61] Bradbury PJ , Zhang Z, Kroon DE et al. TASSEL: software for association mapping of complex traits in diverse samples. Bioinformatics. 2007;23:2633–5. 17586829 10.1093/bioinformatics/btm308

[62] Zhang Z , Ersoz E, Lai CQ et al. Mixed linear model approach adapted for genome-wide association studies. Nat Genet. 2010;42:355–60. 20208535 10.1038/ng.546 PMC2931336

[63] Wang Q , Tian F, Pan Y et al. A SUPER powerful method for genome wide association study. PLoS One. 2014;9:e107684. 25247812 10.1371/journal.pone.0107684 PMC4172578

[64] Liu X , Huang M, Fan B et al. Iterative usage of fixed and random effect models for powerful and efficient genome-wide association studies. PLoS Genet. 2016;12. 10.1371/journal.pgen.1005767 PMC4734661 26828793

[65] Huang M , Liu X, Zhou Y et al. BLINK: a package for the next level of genome-wide association studies with both individuals and markers in the millions. Gigascience. 2019;8. 10.1093/gigascience/giy154 PMC6365300 30535326

[66] Endelman JB . Ridge regression and other kernels for genomic selection with R package rrBLUP. Plant Genome. 2011;4:250–5.

[67] Legarra A , Robert-Granié C, Croiseau P et al. Improved lasso for genomic selection. Genet Res (Camb). 2011;93:77–87. 21144129 10.1017/S0016672310000534

[68] Ogutu JO , Piepho HP, Schulz-Streeck T. A comparison of random forests, boosting and support vector machines for genomic selection. BMC Proc. 2011;5:1–5. 10.1186/1753-6561-5-S3-S11 PMC3103196 21624167

[69] Shikha M , Kanika A, Roa AR et al. Genomic selection for drought tolerance using genome-wide SNPs in maize. Front Plant Sci. 2017;8. 10.3389/fpls.2017.00550 PMC5399777 28484471

[70] Brandenberger L . Studies to quantify disease resistance in spinach to the white rust (*Albugo occidentalis*) and downy mildew (*Peronospora farinosa* f. sp. *spinaciae*) pathogens. In: Doctoral Dissertation. University of Arkansas: Fayetteville, USA, 1992.

[71] Keller B , Ariza-Suarez D, de la Hoz J et al. Genomic prediction of agronomic traits in common bean (*Phaseolus vulgaris* L.) under environmental stress. Front Plant Sci. 2020;11. 10.3389/fpls.2020.01001 PMC7381332 32774338

[72] Qin J , Shi A, Song Q et al. Genome wide association study and genomic selection of amino acid concentrations in soybean seeds. Front Plant Sci. 2019;10. 10.3389/fpls.2019.01445 PMC6873630 31803203

[73] Spindel JE , Begum H, Akdemir D et al. Genome-wide prediction models that incorporate de novo GWAS are a powerful new tool for tropical rice improvement. Heredity (Edinb). 2016;116:395–408. 26860200 10.1038/hdy.2015.113 PMC4806696

[74] Bhattarai G , Shi A. Research advances and prospects of spinach breeding, genetics, and genomics. Vegetable Research. 2021;1:1–18.

